# Argon Laser Peripheral Iridoplasty and Argon Laser Pupilloplasty: Alternative Management for Medically Unresponsive Acute Primary Angle Closure

**DOI:** 10.1155/2019/1876912

**Published:** 2019-08-14

**Authors:** Wenkai Zhou, Fangkun Zhao, Dong Shi, Majida Qadri, Lingfeng Jiang, Liwei Ma

**Affiliations:** ^1^Department of Ophthalmology, The Fourth Affiliated Hospital of China Medical University, Shenyang, China; ^2^Eye Hospital of China Medical University, Shenyang, China; ^3^Key Lens Research Laboratory of Liaoning Province, Shenyang, China; ^4^Tulane University, New Orleans, LA, USA

## Abstract

**Objective:**

To introduce the combined laser technique, argon laser peripheral iridoplasty (ALPI) and argon laser pupilloplasty (ALPP), in the management of medically unresponsive acute primary angle closure (APAC).

**Design:**

Retrospective study.

**Methods:**

We retrospectively reviewed the records of 23 patients (27 eyes) with APAC, who were applied ALPI and ALPP when traditional treatment failed. The visual acuity and intraocular pressure (IOP) were monitored before surgery and at 1, 2, 12, 24, and 48 h after surgery. Additionally, the angle-opening status was monitored before surgery and 48 h after the treatment by using an ultrasonic biological microscope (UBM), and the presurgical and postsurgical cornea edema statuses were observed by using a slit lamp. We also documented the complications of laser treatment.

**Results:**

For the ALPI + ALPP laser-effective group, the presurgical IOP was 52.1 ± 9.3 mmHg and the postsurgical IOP was 37.6 ± 10.9 mmHg (1 h), 28.4 ± 12.4 mmHg (2 h), 19.9 ± 9.0 mmHg (6 h), 16.8 ± 7.3 mmHg (12 h), 15.9 ± 5.9 mmHg (24 h), and 14.9 ± 5.0 mmHg (48 h), with statistically significant differences (*p* < 0.05) in each time point. It was observed in all the patients that the corneal edema alleviated, the angles opened, and visual acuity recovered with varying degrees at 48 h after applying combined laser treatment. For the ALPI + ALPP laser-ineffective group, further interventions were taken. Definite treatment was given in both groups to maintain the long-term IOP control.

**Conclusions:**

Although the combination of ALPI and ALPP is a temporizing therapeutic strategy for APAC, it is effective in relieving pupillary block which is unresponsive to miotic agents, opening the closed angle to a certain extent, restoring the transparency of cornea, and reducing IOP to a safe level for further definitive treatment.

## 1. Introduction

Primary angle-closure glaucoma (PACG) is the second leading cause of irreversible blindness worldwide, particularly in East Asians. It is estimated that there will be 21.82 million patients with PACG in China by the year of 2020 [1]. Patients with PACG attack may present dramatic signs that can lead to blindness if not properly treated. Thus, acute primary angle closure (APAC) requires emergency management of lowering the intraocular pressure (IOP) and resolution of relieving pupil block [2]. Recent studies suggested that multiple factors interplay in the pathogenesis of APAC, among which relative pupillary block is considered to be a significant mechanism for angle closure [3]. Due to pupillary block, the aqueous outflow from the posterior to anterior chamber through the pupil is resisted and thereafter causes the peripheral iris bombe, consequently creating a narrow angle or angle closure [4]. Although laser peripheral iridotomy has been considered as the initial treatment for primary angle closure caused by pupillary block, it does not widen the angle in all cases [5]. It was discovered in the Liwan Eye Study that one-fifth of eyes with suspected APAC had residual angle closure after laser peripheral iridotomy [6]. In these cases, nonpupillary block mechanisms, such as lens-induced, plateau iris and peripheral angle crowding, may be involved. Wang et al. suggested that 54.8% of Chinese PACG is caused by coexisting factors [7].

Conventional treatment of APAC involves medical treatment and laser peripheral iridotomy so as to lower the IOP and relieve pupillary block. Mild attacks may be broken by cholinergic agents (pilocarpine 1-2%), which induce miosis that pulls the peripheral iris away from the trabecular meshwork. However, when the IOP is markedly elevated, the pupillary sphincter may become ischemic and unresponsive to miotic agents alone. Under this circumstance, stronger miotics should be avoided, as they may increase the vascular congestion of the iris or push the lens-iris diaphragm more anteriorly, increasing the pupillary block. Studies have reported that argon laser peripheral iridoplasty (ALPI) is a simple and safe procedure that effectively opens closed angle [8]. This method is useful in reversing APAC attack when antiglaucoma medications fail and corneal edema and shallow anterior chamber preclude immediate laser iridotomy [9]. Like ALPI, argon laser pupilloplasty (ALPP) is another technique which may be used in combination with other procedures such as ALPI to relieve the pupillary block when the pupillary sphincter cramps [10]. Once corneal edema becomes clear enough and IOP is lowered to an adequate level in 48 h, definitive treatment to prevent recurrence of another acute attack must be performed.

In order to facilitate aqueous drainage and relieve pupillary block in APAC, our study adopted ALPI combined with ALPP cooperated with antiglaucoma drug treatment simultaneously so that favorable conditions for the definitive treatment are provided in the next step.

## 2. Materials and Methods

### 2.1. Clinical Data Collection

A total of 23 patients (27 eyes), 8 males (9 eyes) and 15 females (18 eyes), with their first presentation of APAC in the Fourth Affiliated Hospital of China Medical University from Jan 2017 to Oct 2018, were included in this study. Their age ranged from 39 to 82 years (mean age ± standard deviation, 63.2 ± 11.6 years). Inclusion criteria were as follows: (1) patients were diagnosed with APAC with no previous history; (2) the IOP increased in all patients with varying degrees of corneal edema, shallow anterior chamber, mydriasis, and iris bombe; (3) best-corrected vision acuity (BCVA) was from light perception to 0.4 without self-medication or prior intraocular surgical treatment. Exclusion criteria included the following: (1) cornea clear enough for immediate laser peripheral iridotomy; (2) corneal opacity obstructing laser access to the peripheral iris; (3) single-eyed patients.

Patients were administered with a topical treatment of 2% carteolol eye drops, systemic treatment of 50 mg bid methazolamide tablets as oral therapy, and 20% mannitol IV injection. The patients' average IOP was 52.1 ± 9.3 mmHg (IOP was given as 60 mmHg if over the noncontact tonometer testing limit). Data collection conformed to all local laws and was conducted according to the principles of the Declaration of Helsinki. Informed consent was obtained from all patients after explanation of the nature and possible consequences of the study. The study was approved by the Ethics Committee of the Fourth Affiliated Hospital of China Medical University (Registration No. EC-2018-KS-047).

### 2.2. Laser Procedures

All patients underwent a comprehensive ophthalmic examination including refraction, static and dynamic Goldmann gonioscopy, fundus examination and automated perimetry (HAAG-STREIT, Octopus 900) if possible, and ultrasound biomicroscopy (UBM) before treatment. All laser procedures were performed by a senior glaucoma specialist (W. Z.). Multiple wavelength laser (Nidek Co., Ltd., Japan) and contact lens (Abraham) were applied for the laser treatment. (1) Before laser treatment, 0.5% proparacaine was applied as local anesthesia and levofloxacin gel was applied as the corneal contact protective agent. (2) ALPI (420–480 *μ*m spot size, 0.25- to 0.3-second duration, and initially 280–380 mW power) was set to produce contraction burns. With the contact lens in place, the beam is aimed at the extreme peripheral portion of the iris. Treatment consists of placing approximately 20–24 spots over 360°, leaving 1-spot diameter between each spot and avoiding large vessels if possible. The contraction effect is immediate and usually accompanied by noticeable deepening of the peripheral anterior chamber at the site of the burn. Selected energy power should be able to obtain visible stromal contraction but should not produce bubble formation or pigment release into the anterior chamber. (3) ALPP (280–380 *μ*m spot size, 0.2- to 0.25-second duration, 260–320 mW power) was applied to the iris surface of pupillary dilator muscle which is on the lateral side of the sphincter muscle. Not all patients require extensive treatment of the sphincter in order to peak the pupil. Four to six spots were placed in the periphery to achieve pupil peaking, and the aqueous humor outflow from the burn sites should be observed. (4) Postoperative management: continuous application of pranoprofen eye drops (qid), 0.1% fluorometholone eye drops (qid), and 20% mannitol (IV injection) after operation.

### 2.3. Outcome Measurement

IOP values at postoperative 1 h, 2 h, 6 h, 12 h, 24 h, and 48 h were measured. Corneal edema status was recorded according to the modified grading standard of corneal edema by using a slit lamp: Grade 0 represents corneal transparency without edema; Grade 1 displays the corneal localized haze edema, the endothelial layer of the cornea is smooth, and the iris texture is clearly visible; Grade 2 displays the pale gray edema of the cornea, the endothelial layer of the cornea is rough, and the iris texture is blurred; Grade 3 displays diffuse gray edema of the cornea, the corneal endothelium is cracked, and the iris is unclear; Grade 4 displays the ivory edema of the cornea, and the intraocular structure is not clear. The original corneal opacity grading criteria were firstly introduced by Wang et al. in their paper about congenital glaucoma [11]. Status of the anterior chamber was measured by using an UBM. Images were obtained, and the configuration of the iris, the depth of the corneal anterior chamber, and appositional angle closure were evaluated. Meanwhile, visual acuity at 48 h after operation and other intraoperative or postoperative complications were observed.

### 2.4. Statistical Analysis

Statistical analysis was carried out using SPSS 17.0 software package. The paired *t*-test was applied for testing the significance of each group. Data were expressed as mean ± SD, with *p* < 0.05  considered as statistically significant.

## 3. Results

### 3.1. Workflow of Treatment Process

**Figure 1:**
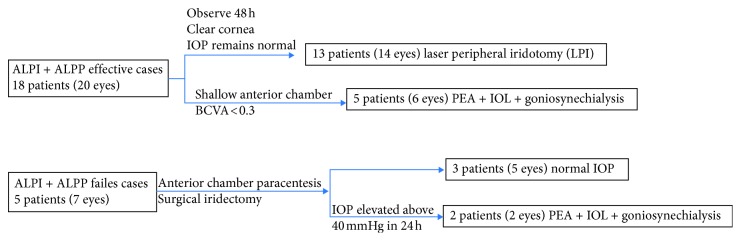
Interventions taken in the treatment process. Effective cases: IOP decreases in 2 h after the laser procedure and remains normal in 24 h. Failed cases: IOP > 40 mmHg in 6 h after the laser procedure.

IOP was measured at different time points before and after laser therapy. If IOP began to decrease in 2 h after the laser procedure and reached normal range in 24 h, it was considered as effective treatment. If IOP remained at the high level (>40 mmHg) at 6 h after the laser procedure, it was considered as failed treatment. The treatment process was shown to demonstrate the interventions applied on different patients (Figure 1). As shown in Table 1, ALPI and ALPP worked effectively in 18 patients (20 eyes) and failed in 5 patients (7 eyes).

### 3.2. Results of the Effective Cases

#### 3.2.1. Intraocular Pressure Changes before and after Surgery

**Figure 2:**
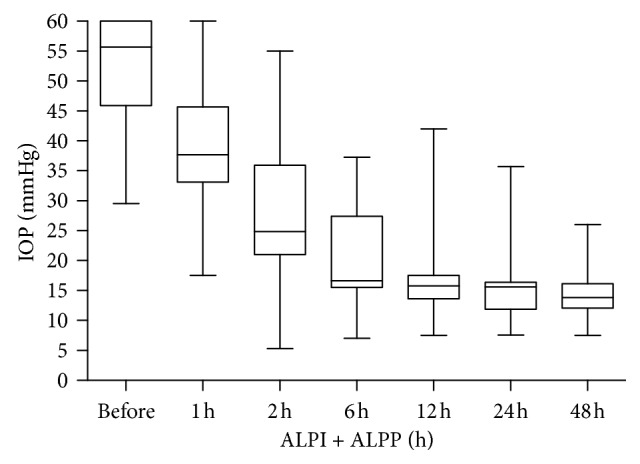
Time course of IOP before and after surgery.

IOP was reduced from 52.1 ± 9.3 mmHg (before laser therapy) to 37.6 ± 10.9 mmHg (1 h), 28.4 ± 12.4 mmHg (2 h), 19.9 ± 9.0 mmHg (6 h), 16.8 ± 7.3 mmHg (12 h), 15.9 ± 5.9 mmHg (24 h), and 14.9 ± 5.0 mmHg (48 h) after laser therapy (Figure 2). The paired *t*-test was used to analyze the differences of pre- and post-laser therapy IOP of the patients, and a *p* value less than 0.05 was considered to be statistically significant (Table 2).

#### 3.2.2. Best-Corrected Visual Acuity (BCVA) before and after Surgery

Preoperative BCVA ranged from light perception to 0.5. Eight eyes were between light perception and 0.05, which accounted for 40%; seven eyes were between 0.05 and 0.1, which accounted for 35%; three eyes were between 0.1 and 0.3, which accounted for 15%; and two eyes were greater than 0.3, which accounted for 10%. Postoperative BCVA at 48 h ranged from 0.1 to 0.8. Six eyes were between 0.1 and 0.3, which accounted for 30%; fourteen eyes were greater than 0.3, which accounted for 70% (Table 3).

#### 3.2.3. Slit-Lamp Examination on Corneal Edema Grade Assessment

The grading system for corneal edema is helpful in evaluating the severity of endothelial damage and can be used to predict the outcome of the laser surgery. Corneal edema in grades 1, 2, and 3 accounted for 10%, 30%, and 60% before surgery. Postoperative corneal edema was observed to be decreased after 48 h with grades 0 and 1 which accounted for 50% and 50%, respectively (Table 4). The iris bombe was observed to be disappeared or decreased, mydriasis was also observed with varying degrees, and pupillary light reflex was decreased in some and absent in the other objectives.

#### 3.2.4. Gonioscopy and UBM Examination

**Figure 3:**
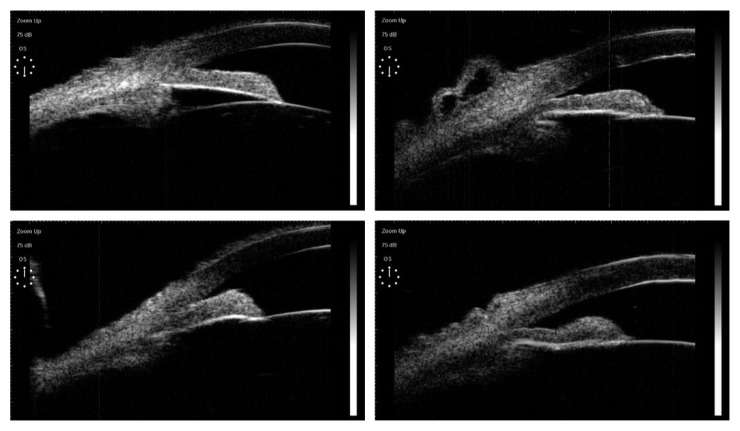
Comparison of angle configuration of the effective cases (left: before surgery and right: after surgery).

Since 6 patients (6 eyes) required further intraocular surgeries after 48 h, angle examination was not performed on these 6 patients. The angle-opening status of 9 eyes was greater than 180 degrees, which accounted for 64.3%. Four eyes were observed with an angle opening between 90 and 180 degrees, which accounted for 21.4%. Two eyes were observed having an angle opening of less than 90 degrees, which accounted for 14.3% (Table 5). The comparison of angle configuration of effective cases is shown in Figure 3. The midperipheral iris was thinner due to the laser burn, and the angle was opened by the contraction of the iris root, with the aqueous humor in the posterior chamber flew flowing through the pupil peak into the anterior chamber, and the iris bombe flattened.

#### 3.2.5. Intraoperative and Postoperative Complications

One eye was observed with mild corneal endothelial injuries with no additional treatment required. Local and mild inflammation was observed and treated with eye drops containing steroids. Fundus decompression hemorrhage was observed in one eye, and with oral administration of drugs promoting absorption and circulation, this patient was treated as retinal hemorrhage after the glaucoma stabilization. Pupil dilation and deformation were observed after laser treatment in all of the patients. Light reflex was disappeared in some cases.

### 3.3. Further Interventions with Patients

#### 3.3.1. Effective Cases

After IOP normalized for 48 hours and cornea became clear enough, laser peripheral iridotomy (LPI) was performed in 13 patients (15 eyes). For the other 5 patients (5 eyes) whose anterior chambers were still shallow and BCVA values were less than 0.3, phacoemulsification plus intraocular lens implantation was performed and followed with goniosynechialysis by using ophthalmic viscosurgical devices (OVD). With these further interventions, all the 18 patients (20 eyes) presented normal IOP during follow-up till now.

#### 3.3.2. Failed Cases

**Figure 4:**
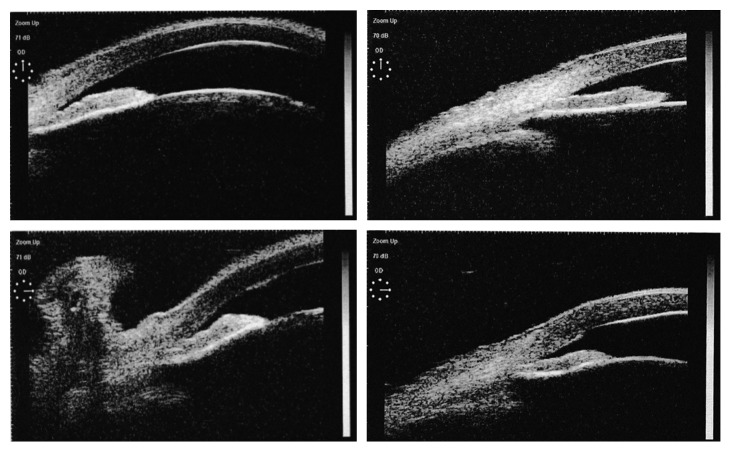
Comparison of angle configuration of the failed cases (left: before surgery and right: after surgery).

In order to minimize the optical damage due to the extremely elevated IOP, anterior chamber paracentesis and surgical iridectomy were performed in the 5 patients (7 eyes) whose IOP remained at a high level at 6 h after ALPI and ALPP. In the 2 patients (2 eyes) whose IOP elevated above 40 mmHg again in 24 h, phacoemulsification plus intraocular lens implantation was performed and followed with goniosynechialysis by using OVD. With these surgical interventions, all the 5 patients (7 eyes) presented normal IOP during follow-up till now. The comparison of angle configuration of the failed cases is shown in Figure 4. The lens and iris contacted extensively because of the higher lens vault. The laser burn of ALPP was not powerful enough to break the pupil block to facilitate the aqueous humor flow. Though there was midperipheral iris thinning due to the laser burn, the anterior chamber was still shallow and the angle remained closed.

## 4. Discussion

APAC occurs when IOP rises rapidly as a result of relatively sudden blockage of the trabecular meshwork by the peripheral iris bombe. It is typically manifested by ocular pain, headache, blurred vision, rainbow-colored halos around lights, nausea, and vomiting. The management of APAC is directed at lowering the IOP and relieving pupil block. The conventional treatment to lower the IOP includes topical and systemic medical therapies, which may allow resolution of IOP-induced corneal edema and enable subsequent definitive treatment by relieving pupil block typically using laser peripheral iridotomy [12]. However, the relatively high level of IOP may produce sector atrophy of the iris due to ischemia and such atrophy could release pigment and cause pigmentary dusting of the iris surface and corneal endothelium. Ischemia of the iris sphincter muscle may also cause the pupil fixed and dilated permanently; thus, medications cannot achieve obvious effect because the pupil muscle cramp even could aggravate pupillary block factors. In order to prevent the recurrence of another acute attack, a surgical iridectomy or, more commonly, laser peripheral iridotomy should be accomplished once the attack is broken and the cornea is of adequate clarity. Due to the noninvasive nature and ease of performing, laser peripheral iridotomy is considered to be a therapeutic solution for APAC. The advantage of this surgical method is that it can link up the anterior and posterior chamber and reduce the pupil blockage. However, due to the corneal edema, visibility of the anterior chamber declined seriously and laser peripheral iridotomy may be precluded and is prone to cause complications including hyphema and monocular blurring.

Emerging strategies that lower IOP include anterior chamber paracentesis, as well as laser procedures such as iridoplasty and pupilloplasty. The peripheral iris may be flattened with a laser iridoplasty or the pupillary block may be relieved with a laser pupilloplasty [9, 13]. Anterior chamber paracentesis is an alternative method of rapidly reducing IOP and being repeatable [12]. However, this procedure must be undertaken with great caution. The rapid decompression can result in a suprachoroidal hemorrhage or hyphema. Meanwhile, this is an intraocular procedure, and endophthalmitis may also occur. Considering the mixed mechanism of APAC, it made clinical sense to try a combined iridoplasty and pupilloplasty approach to reduce the IOP, relieve pupillary block, and obtain clear corneal edema. An attack of APAC that is unresponsive to medical therapy and in which corneal edema, shallow anterior chamber, or marked inflammation preclude laser iridotomy may be broken with ALPI [14]. ALPI does not acquire high visibility of the anterior chamber. In our study, ALPI was performed without any difficulty to all the included 27 eyes, in which LPI was precluded because of the edematous cornea. ALPI involves applying laser burns to the peripheral iris, causing contraction of the iris stroma and mechanically pulling the angle open, hence facilitating trabecular aqueous drainage [5]. As a viable alternative first-line treatment for APAC, it reduces IOP more rapidly than medications in the first few hours. A randomized controlled trial was designed to explore the efficacy and safety of LPI with or without ALPI in the treatment of PAC with multiple mechanisms [15]. Currently, opinion is that laser peripheral iridotomy alone is not sufficient to prevent disease progression. However, there was no significant difference in IOP, medications, need for surgery, or visual function between groups at the 1-year visit [5].

For pupillary block acute angle-closure attack, mydriatic drugs should be considered to reduce the gap resistance between the iris and lens. However, due to high IOP, the sphincter muscle is cramped, causing the pupil unresponsive to the medications. Under this circumstance, conducting laser pupilloplasty simultaneously by adopting interval laser burns can cause the contraction of pupil dilator muscle and pupil peaking is achieved. With the IOP gradient in the posterior chamber, the aqueous humor may flow into the anterior chamber, resulting in the angle opening of the anterior chamber, increase in drainage, and reduction in IOP. In such situation, ALPP worked similarly as LPI which facilitates the communication between the posterior and anterior chamber. In this study, the effective rate of the combined procedures was 74.1% (20/27). The effect of the laser interventions on the configuration of the anterior segment was proven by the pre- and post-photocoagulation UBM examination (Figure 3).

However, the combined ALPI and ALPP procedures did not work in all of the cases. In the 7 eyes failed in reducing IOP, the configuration of anterior segment of these eyes seemed to be of shallower anterior chamber depth, higher lens vault, and extensive contact area of the iris and lens. It was a pity that the statistical analysis was not performed due to the smaller number of cases and the inaccuracies of the manual calibration of UBM. The contraction caused by the ALPI laser burn was not powerful enough to pull the iris root away from the closed angle, and the contraction caused by the ALPP laser burn was not enough to achieve pupil peak because of the extensive contact area of the iris and lens (Figure 4).

The attack of APAC is usually of multiple mechanisms, and the ALPI mainly works in the presence of plateau iris, while ALPP works when pupillary block is present. That is why we applied the combined ALPI and ALPP procedures in this series of cases. However, the IOP-lowering effect of the combined procedures was rarely permanent because of their mechanism. When IOP remained normalized and cornea returned clear, in our cases, in 48 hours after the laser intervention, LPI was performed for definite communication of the anterior and posterior chamber in 15 eyes. For the other 5 eyes whose anterior chamber was still shallow and BCVA was less than 0.3, with high possibility of relapse, phacoemulsification plus intraocular lens implantation was performed and followed with goniosynechialysis by OVD. Surgical peripheral iridotomy, then phacoemulsification plus intraocular lens implantation followed with goniosynechialysis by OVD, if necessary, was applied to the failed cases as well. The IOP of all the cases was in normal range during follow-up. Anyhow, the purpose of APAC treatment is to control the IOP as soon as possible by all available means to minimize the damage on the optic nerve.

Currently, there is no observational study comparing the efficacy and safety between ALPI with or without ALPP in APAC patients. In this study, the IOP can be controlled to a safe level within 48 h in 74.1% of the cases by the combined laser procedures, which gained time for further definite treatment to control IOP. Although the combination of ALPI and ALPP is a temporizing measurement to lower the IOP, it might become a potential surrogate to replace the traditional anterior chamber paracentesis and drug therapy because of its safety and efficacy in the future.

## Figures and Tables

**Table 1:** Outcome of ALPI and ALPP on patients (eyes).

	Effective cases	Failed cases	Total
Male (eyes)	7 (7)	1 (2)	8 (9)
Female (eyes)	11 (13)	4 (5)	15 (18)
Total	18 (20)	5 (7)	23 (27)

**Table 2:** Preoperative and postoperative IOP (mean ± SD, mmHg).

Before	After 1 h	After 2 h	After 6 h	After 12 h	After 24 h	After 48 h
52.1 ± 9.3	37.6 ± 10.9	28.4 ± 12.4	19.9 ± 9.0	16.8 ± 7.3	15.9 ± 5.9	14.9 ± 5.0
*t*	4.526	6.821	11.13	13.37	14.69	15.71
*p*	<0.05	<0.05	<0.05	<0.05	<0.05	<0.05

**Table 3:** Preoperative and postoperative BCVA (*n* (%)).

	<0.05	0.05–0.1	0.1–0.3	≥0.3
Before	8 (40%)	7 (35%)	3 (15%)	2 (10%)
After	0 (0%)	0 (0%)	6 (30%)	14 (70%)

**Table 4:** Preoperative and postoperative corneal edema grade (*n* (%)).

	Grade 0	Grade 1	Grade 2	Grade 3	Grade 4
Before	0 (0%)	2 (10%)	6 (30%)	12(60%)	0 (0%)
After	10 (50%)	10 (50%)	0 (0%)	0 (0%)	0 (0%)

**Table 5:** Postoperative angle-opening degree (*n* (%)).

	>180	90–180	<90	Total
Angle-opening degree	9 (64.3%)	3 (21.4%)	2 (14.3%)	14

## Data Availability

The images and data used to support the findings of this study are included within the article.
